# Interparental Conflict Relative to Suicidal Ideation in Chinese Adolescents: The Roles of Coping Strategies and Meaning in Life

**DOI:** 10.3389/fpsyg.2017.01010

**Published:** 2017-06-22

**Authors:** Rongwei Zhang, Dan Li, Fei Chen, Béatrice M. Ewalds-Kvist, Shihong Liu

**Affiliations:** ^1^Department of Psychology, Shanghai Normal UniversityShanghai, China; ^2^Department of Social Science, Fujian Polytechnic of Information TechnologyFuzhou, China; ^3^Department of Anesthesiology, The University of North Carolina at Chapel Hill, Chapel HillNC, United States; ^4^Department of Psychology, Stockholm UniversityStockholm, Sweden; ^5^Department of Psychology, Turku UniversityTurku, Finland

**Keywords:** adolescents, coping strategies, interparental conflict, meaning in life, serial mediation, suicidal ideation

## Abstract

The aim of this study was to explore the paths between interparental conflict and Chinese adolescents’ suicidal ideation. Altogether 931 adolescents (*M_age_* = 17.84, *SD* = 0.77, females = 531) completed the Dyadic Consensus Scale, Self-Report Coping Scale, Meaning in Life Questionnaire, and Positive and Negative Suicide Ideation questionnaires. Mediation analyses were conducted, focusing on the relations between interparental conflict and suicidal ideation along with coping styles and a sense of meaning in life. The results showed that interparental conflict indirectly predicted adolescents’ suicidal ideation via three mediators: coping-approach strategies, presence of meaning, and the joint serial effects of coping-approach strategies and presence of meaning in Chinese adolescents. In addition, boys were more likely to be at risk for suicidal ideation than girls, so were 10th graders compared to 11th graders. These findings supported a combined distress-to-meaninglessness line of thinking along with the use of coping-approach strategies to depress self-harm ideation. Generally, interparental conflict should be kept out of youngsters’ immediate vicinity as a preventive measure of suicidal ideation.

## Introduction

China has in recent decades experienced a remarkable economic growth (Nolan, 2007). But, in contrast, the country’s non-material facet of well-being has been somewhat neglected (Mok et al., 2010; Shek, 2010); a fact that comes to light in adolescents’ escalating numbers of suicides (Phillips et al., 2002; Shah, 2007). The prevalence of suicides is 10.72% in Chinese students (Li et al., 2014; Tang and Qin, 2015) and self-harm ideation is a precursor to attempted or completed suicide (Zhang et al., 2012; Burke and Alloy, 2016). While suicidal behavior is frequently related to the presence of psychiatric conditions, particularly mood disorders (Pompili et al., 2009) we studied students in real life conditions with no – to our knowledge – known psychiatric history. Furthermore, coping strategies have been found to predict mental quality of life (Engel-Yeger et al., 2016). Thus, self-harm ideation relative to interparental conflicts and coping tactics are presently in the focus of interest.

This study explored the paths from interparental conflict to suicidal ideation inspired from a distress-to-meaninglessness line of thinking. The conceptual model is based both on the stress and coping idea by Lazarus (1966) as well as on the emotional security theory by Davies and Cummings (1994). This combined conceptual model emphasizes the role of meaningfulness and addresses the process by which an adolescent’s suicidal ideation is shaped. Coping failure with interparental conflict increases psychological distress and a sense of life’s meaninglessness which predicts self-harm ideation (Li et al., 2012; Bergman et al., 2014; Wang et al., 2014). Specifically, interparental conflict is known to increase adolescents’ maladaptive coping behavior (Shelton and Harold, 2007, 2008) or to decrease adolescents’ adaptive coping behavior (Wadsworth and Compas, 2002). Interparental conflict also threatens adolescents’ emotional security (Cummings and Davies, 1996) as opposed to a sense of life’s meaningfulness, which buffers against distress and self-harm (Kiang and Witkow, 2015). The term meaningfulness comprises both presence of and/or search for meaning (Frankl, 1985) which may serve as mediator or moderator, respectively, between distress and self-harm (Marco et al., 2016). As a mediator, decreased presence of meaning explains that despair leads to self-harm; as a moderator, presence of meaning buffers the effect of despair on suicidal ideation (Henry et al., 2014). Presence of meaning occurs independently of search for meaning (Datu and Mateo, 2015); search may diverge from presence of meaning as a mediator or moderator in the relationship between interparental conflict and suicidal ideation. Further, meaningfulness buffers also between coping style and self-harm (Winter et al., 2010). Coping strategy *per se* predicts mental quality of life (Engel-Yeger et al., 2016). Therefore, the pathways between interparental conflict and suicidal ideation may bridge over in two ways: first, through coping strategies and second, through a sense of meaning in life. Consequently, we scrutinized if interparental conflict relates to adolescents’ suicidal ideation in three ways: does interparental conflict alter youngsters’ coping strategies? Does interparental conflict change adolescents’ sense of presence of or search for meaning in life? Does a joint serial effect of altered coping strategies along with an altered sense of presence of or search for meaning relative to interparental conflict, exist in young people’s life?

## Materials and Methods

### Participants

Participants were 931 adolescents (400 males, 531 females) from grades 10 (*n* = 554) and 11 (*n* = 377) ranging in age from 16 to 21 years (*M_age_* = 17.84, *SD* = 0.77). All participants were healthy and with no known psychiatric history. Most of them (*n* = 858, 92.2%) were not known to have experienced stressful event such as serious illness. A few participants (*n* = 7, 0.8%) had experienced stressful family events such as illness or death of grandparents, parental job change, and frequent family relocation. Some participants (*n* = 66, 7%) did not report if they had experienced stressful family events. Most of the participants (*n* = 913, 98.1%) have intact family and 0.9% (*n* = 8) participants lived in divorced or remarried families. One percent (*n* = 10) of the parents did not reveal the status of marriage. More than 93% of the parents had at least middle school education. Altogether 418 (44.9%) fathers and 364 (39.1%) mothers were educated on an undergraduate degree or above level. Most of the participants were from middle-income families in China. Participants were recruited by means of cluster sampling method from three high schools in Shanghai, China.

### Measures

#### Dyadic Consensus Scale (DCS)

Both parents of the participating adolescents completed the Dyadic Consensus Scale (DCS), a subscale of Dyadic Adjustment Scale (DAS, Spanier, 1976), which measures marital conflicts. The DCS comprises eight items involving conflicts on parenting, income, relations with partner’s parents and relatives, friendship, pleasures, affectional discord, household duties, attitudes to something, etc. All items were rated on a four-point scale (from 1 = always to 4 = never). Higher scores represented less severe interparental conflict. DAS has been widely used in Chinese samples and proved to be of good reliability and validity (Shek, 1995; Lim and Ivey, 2000). In this study, the internal reliabilities were 0.84 for mothers and 0.85 for fathers.

#### Self-Report Coping Scale (SCS)

The Self-Report Coping Scale (SCS) (Causey and Dubow, 1992), revised by Kingsbury et al. (2014), consists of a 34-item self-report measure of coping strategies. It comprises five subscales: help-seeking, problem-solving, internal behavior (e.g., worry too much about it), external behavior (e.g., cursing loudly) and avoidance (e.g., forgetting the whole thing). Help-seeking and problem-solving strategies belong to coping-approach strategies and the other three subscales constitute coping-avoidant strategies. The items are rated from 1 (never) to 5 (always). Higher scores suggest that more of these coping strategies are in use. The reliability and validity of the SCS have been reported in previous research (Kingsbury et al., 2014). In this study, the internal reliabilities were 0.91 for the subscale of coping-approach strategies, 0.92 for the subscale of coping-avoidant strategies, and 0.89 for the whole scale.

#### The Meaning in Life Questionnaire (MLQ)

The Meaning in Life Questionnaire (MLQ) (Steger et al., 2006) included two subscales: presence of meaning and search for meaning. Each subscale comprises five items. All items range from 1 (completely false) to 7 (completely true). Higher scores indicate a stronger sense of meaning in life. The MLQ has acceptable reliability and validity in Chinese sample (Liu and Gan, 2010). Internal reliabilities were 0.84 for ‘presence of meaning,’ 0.83 for ‘search for meaning,’ and 0.79 for the total scale in the present study.

#### Positive and Negative Suicide Ideation (PANSI)

The Positive and Negative Suicide Ideation (PANSI) (Osman et al., 1998) assessed the frequency of risk and protective factors relative to adolescents’ suicidal ideation. It includes a six-item subscale of positive suicidal ideation and an eight-item subscale of negative suicidal ideation. Each item is rated on a five-point scale ranging from 1 (none of the time) to 5 (most of the time). The subscale of positive suicidal ideation is reversely scored – such that higher scores are indicative of stronger suicidal ideation. The scale has been shown to have high reliability and validity in China (Chang et al., 2007, 2009). The internal reliabilities were 0.87 for the subscale of positive suicidal ideation, 0.95 for the subscale of negative suicidal ideation, and 0.87 for the total scale in the present study.

### Procedure

Ethical procedures were followed throughout the study. The Shanghai Normal University and schools’ institutional review board approved the study in advance of data collection. Prior to data collection, informed consent was obtained from participants and their parents, and a brief instruction about truth and privacy were emphasized by a trained graduate researcher. A multi-informant method was used. Data were collected from both students and their parents. Students from the same class were assembled in the school computer room where they completed the online survey. Parents’ questionnaires were brought home by students to be completed by their fathers and mothers. Students brought back the parents’ questionnaires to school in a sealed envelope and returned them to their teachers. Students were rewarded with a small gift (e.g., a pen) for their participation.

### Statistical Analysis

A multivariate analysis of variance (MANOVA) was conducted to examine the overall effects of gender, grade, and their interactions on interparental conflict, meaning in life, coping strategies, and suicidal ideation. Correlation analyses were used to examine the associations among all the study variables. Based on correlations of the variables, mediation analysis, a regression-based approach, was constructed to examine the hypothesized mediation models. The collected data were analyzed using IBM SPSS Statistics version 22.0 and macro-program PROCESS 2.1 (Hayes, 2013).

## Results

### Descriptive Statistics

Findings from MANOVA revealed significant main effects of gender, *Wilks’ λ =* 0.95, *F*(6,922) = 8.51, *p* < 0.001, η^2^ = 0.05, and grade, *Wilks’ λ =* 0.98, *F*(6,922) = 2.48, *p* < 0.05, η^2^ = 0.02. There was no significant gender × grade interaction effect, *Wilks’ λ =* 0.99, *F*(6,922) *= 0.82, p* > 0.05, η^2^ = 0.01. Results from univariate tests revealed significant gender differences on suicidal ideation and approaching coping strategies, *F*(1,927) = 31.10, *p* < 0.001, η^2^ = 0.03, *F*(1,927) = 29.18, *p* < 0.001, η^2^ = 0.03, respectively, and grade differences on interparental conflict and suicidal ideation, *F*(1,927) = 4.97, *p* < 0.05, η^2^ = 0.01, *F*(1,927) = 6.52, *p* < 0.05, η^2^ = 0.01, respectively. *Post hoc* test using Scheffé method indicated that, compared to girls, boys reported higher scores for suicidal ideation, and lower scores for coping-approach strategies. As well, students in 11th grade had higher scores on interparental conflict, but lower scores on suicidal ideation than did students in 10th grade. Means and standard deviations of the variables for boys and girls in each grade are presented in **Table 1**.

**Table 1:** Mean (and standard deviations) of variables for boys and girls.

	Grade 10		Grade 11	
	Boys	Girls	Boys	Girls
Interparental conflicts	3.48 (0.96)	3.48 (0.88)	3.61 (1.02)	3.63 (1.04)
Coping-App Strategies	6.15 (1.35)	6.62 (1.24)	6.18 (1.45)	6.66 (1.21)
Coping-Avo Strategies	8.00 (2.10)	7.66 (1.68)	7.65 (1.93)	7.62 (1.60)
Presence of MIL	4.71 (1.29)	4.71 (1.28)	4.57 (1.38)	4.73 (1.36)
Search for MIL	5.11 (1.33)	5.31 (1.11)	5.15 (1.28)	5.27 (1.25)
Suicidal ideation	2.31 (0.69)	2.00 (0.63)	2.14 (0.71)	1.95 (0.59)

*Coping-App Strategies, Coping-Approach Strategies; Coping-Avo Strategies, Coping-Avoidant Strategies; MIL, Meaning in life.*

Intercorrelations among the studied variables are presented in **Table 2**. Interparental conflict was significantly and negatively correlated with coping-approach strategies (*r* = -0.076) and presence of meaning (*r* = -0.086), significantly and positively related to suicidal ideation (*r* = 0.072), but not significantly associated with coping-avoidant strategies (*r* = -0.003) and search for meaning (*r* = -0.042). Coping-approaching strategies were significantly and positively correlated with presence of meaning (*r* = 0.248) and search for meaning (*r* = 0.196), and significantly and negatively related to suicidal ideation (*r* = -0.431). Both presence of meaning and search for meaning were also significantly and negatively related to suicidal ideation (*r* = -0.308; *r* = -0.108, respectively). The following analyses of hypothesized models were conducted based on the correlation models of variables.

**Table 2:** Associations among variables.

	1	2	3	4	5	6
1 Interparental conflicts	1					
2 Coping-App Strategies	–0.076^∗^	1				
3 Coping-Avo Strategies	–0.003	0.352^∗∗^	1			
4 Presence of MIL	–0.086^∗∗^	0.248^∗∗^	–0.010	1		
5 Search for MIL	–0.042	0.196^∗∗^	0.056	0.141^∗∗^	1	–
6 Suicidal ideation	0.072^∗^	–0.431^∗∗^	0.265^∗∗^	–0.308^∗∗^	–0.108^∗∗^	1

*^∗^*p* < 0.05, ^∗∗^*p* < 0.01.*

### Mediation Model with Coping-Approach Strategies and Presence of Meaning

The correlation analyses revealed that coping-avoidant strategies were not significantly associated with interparental conflict, presence of meaning and search for meaning. Therefore, this variable was not included in the subsequent mediation effect analyses. Consequently, the idea of coping-avoidant strategies as an individual mediator or as joint serial mediator with meaning in life in the relationship between interparental conflict and adolescents’ suicidal ideation was not supported in this study.

**FIGURE 1:**
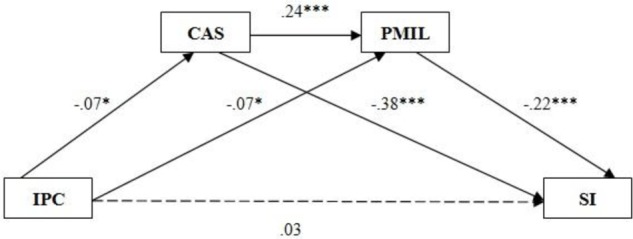
Associations between interparental conflict and suicidal ideation: serial mediation analysis: coping-approach strategies and presence of meaning as serial mediators. IPC, Interparental Conflicts; CAS, Coping-Approach Strategies; PMIL, Presence of Meaning in Life; SI, Suicidal Ideation.

First, we examined whether coping-approach strategies along with presence of meaning serve both as individual mediators as well as joint serial multiple mediators in the relationship between interparental conflict and adolescents’ suicidal ideation, and formulated the first model (**Figure 1**). The results indicated that interparental conflict did not directly predict suicidal ideation (β = 0.03), but it did indirectly predict suicidal ideation in three ways (total indirect effects: 0.05): via the mediator of coping-approach strategies, via the mediator of presence of meaning, and via the serial multiple mediators of coping-approach strategies along with a sense of presence of meaning (**Figure 1**). However, the results supported the line of thinking that coping-approach strategies and presence of meaning serve both as individual mediators and jointly as serial mediators in the relationship between interparental conflict and adolescents’ suicidal ideation.

### Mediation Model with Coping-Approach Strategies and Search for Meaning

**FIGURE 2:**
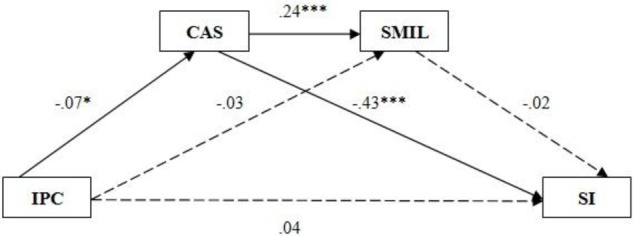
Associations between parental conflicts and suicidal ideation: serial mediation analysis: coping-approach strategies and search for meaning as serial mediators. IPC, Interparental Conflicts; CAS, Coping-Approach Strategies; SMIL, Search for Meaning in Life; SI, Suicidal Ideation.

Second, we explored whether coping-approach strategies and search for meaning serve both as individual mediators as well as serial multiple mediators between interparental conflict and adolescents’ suicidal ideation, and formulated the second model (**Figure 2**). To investigate this possibility, we examined whether search for meaning functions in the same way as presence of meaning, so previously used testing methods and steps were replicated. The result showed that interparental conflict did not directly predict suicidal ideation (β = 0.04) along with search for meaning (β = -0.03); search for meaning did not predict suicidal ideation (β = -0.02) in this model (see **Figure 2**). A model of coping-approach strategies along with search for meaning in life as serial multiple mediators in the relations between interparental conflict and adolescents’ suicidal ideation, was not supported. In contrast, the theory of coping-approach strategies as a mediator in the relation between interparental conflict and adolescents’ suicidal ideation was supported in the second model.

## Discussion

We presently revealed that interparental conflict indirectly affects adolescents’ suicidal ideation through three mediators: coping-approach strategies, sense of presence of meaning, and joint serial multiple mediators first by coping-approach strategies and then by sense of presence of meaning. The role of the sense of presence of meaning in a combined distress-to-meaninglessness line of thinking along with the use of coping-approach strategies to depress self-harm ideation was emphasized and thereby expanded Lazarus (1966) stress and coping model. Interparental conflict constitutes one of the major stressful events for adolescents (Cummings et al., 2002; Xing et al., 2010; Lin et al., 2014) while it clashes with youngsters’ need for security and harmony. It thereby contributes to a sense of meaninglessness in life (Winter et al., 2010; Cummings and Schatz, 2012) and bears predictive power for risks of suicidal ideation among young people (You et al., 2014; Datu and Mateo, 2015).

Interparental conflict predicted also suicidal ideation through the joint serial multiple mediators of coping-approach strategies along with presence of meaning in agreement with findings by Wadsworth and Compas (2002) who indicated that more stressed adolescents used potentially less helpful coping strategies. Yet, they were also more likely to feel less meaning in life and more inclined for suicidal ideation or self-harming behavior (Li and Zhang, 2012). Currently, no relationship between interparental conflict and coping-avoidant strategies was revealed. In other words, adolescents did not use potentially damaging coping strategies when faced with interparental conflict, in disagreement with findings by Wadsworth and Compas (2002). Furthermore, search for meaning was now unrelated to interparental conflict although a sense of presence of meaning in life linked to the negative event. However, it is known that the two meaning components can be inversely related (Steger et al., 2008). Furthermore, Baumeister et al. (2013) made known that unhappy people can live meaningful lives and happy people can live meaningless lives (cf. Ewalds-Kvist and Lützén, 2015). The former spends more time thinking about the past, present and future, as opposed to latter people who is more present oriented and living in the now.

About gender differences, compared with boys, girls scored lower on suicidal ideation, and higher on coping-approach strategies. This disagrees with findings that females who use passive coping strategies were more likely to report suicidal ideation than males (Yao et al., 2014). In China, in big cities, girls accomplish better than boys in academics as well as show more adaptive behaviors and social competence in elementary and middle school (Chen and French, 2008). As a general rule, girls cope better, suffer less from psychological distress and suicidal ideation (Chen et al., 2000; Zhang et al., 2011) in agreement with our present findings. In addition, students in grade 11, compared to those in grade 10, scored higher on interparental conflict but lower on suicidal ideation in agreement with results by Lee and Chan (1993).

Our results indicated that parents’ destructive conflicts harm adolescents’ coping-approach strategies and sense of meaningfulness. Family stress or instability are risk factors in mentally vulnerable individuals who commit suicide (cf. Pompili et al., 2007). The negative effects of interparental conflicts may be further strengthened in the presence of childhood abuse / maltreatment experiences (Pompili et al., 2011). Therefore, family therapy is recommended to reduce interparental conflicts but then again adolescents must also be taught how to handle their own inevitable so called normal conflicts in life (Montemayor, 1983). Yet, the use of coping-approach strategies (Zhang et al., 2014) and meaning making (Park and Folkman, 1997) are effective ways to cope with unexpected stressors; and a coping strategy *per se* may predict mental quality of life (Engel-Yeger et al., 2016). Therefore, by arranging special mental coping training sessions along with support groups for adolescents, feelings of meaninglessness can be prevented. Noteworthily, seeking help is useful in some but not in all situations (Zhang et al., 2014) and coping-avoidant skills (e.g., distancing, fantasizing) are not at all times a dysfunctional way to cope with stressors (Zhang et al., 2012). In sum, adolescents’ learning of adaptive coping skills lessens distress, and maintains a sense of meaningfulness in life.

### Limitations and Future Directions

Although the pathways from interparental conflict to adolescents’ suicidal ideation were presently revealed, this study has its limitations. We did not interview the students and the results are based on their written answers. This means that we were not able in other ways to objectively assess the students’ inclinations toward self-harm. In future studies, also interviews can be applied comprising symptoms, treatments and treatment adherence while attitudes and beliefs predict treatment non-adherence (Pompili et al., 2009), an aspect we did not focus on in this study. Also a possible history of neglect or abuse should be clarified (Pompili et al., 2011). Hitherto, cross-sectional design was used but a longitudinal study could be beneficial in future research. The results are now based on Chinese high-school students, which may or may not generalize to other cultures and other age groups. Future research is warranted to replicate the study in other cultures and thereby extend the generalizability of the results at hand; sequels of interparental conflicts and use of coping strategies could also be explored in different age groups.

## Conclusion

This study provided new insights into the paths between interparental conflict and adolescents’ suicidal ideation. Interparental conflict did not activate suicidal ideation through calling into action coping-avoidant strategies along with presence or search for meaning, however, it did so by calling upon coping-approach strategies along with a sense of meaningfulness in life in the current serial multiple mediation analysis. Gender and grade differences in suicidal ideation were found. Boys were more likely to be at risk for suicidal ideation than girls, and so were students in 10th grade compared to those in the 11th grade. Generally, interparental conflict produces severe stress in adolescents and should be kept out of youngsters’ immediate vicinity.

## Author Contributions

RZ wrote the first draft of the manuscript and assisted in study design, data collection, and data analyses. DL was the principal investigator of the study and led the study. FC revised the manuscript. BE-K wrote part of the first draft of the manuscript, revised and polished it. SL assisted in data collection. All of the authors participated in the final approval of the version to be published and agreed to be accountable for all aspects of the work.

## Conflict of Interest Statement

The authors declare that the research was conducted in the absence of any commercial or financial relationships that could be construed as a potential conflict of interest.
